# Effects of Galilean Loupes on Dental Students' Posture and Cavity Preparation Quality

**DOI:** 10.1111/eje.70010

**Published:** 2025-07-03

**Authors:** Júlia M. Pazos, Matheus M. Canonici, Patricia P. N. S. Garcia

**Affiliations:** ^1^ Department of Social Dentistry São Paulo State University (Unesp), School of Dentistry Araraquara São Paulo Brazil

**Keywords:** dental students, Galilean loupes, quality of the procedure, working posture

## Abstract

**Objectives:**

This study investigated the effect of different magnification levels of the Galilean system loupes on dental students' working posture and the quality of cavity preparations.

**Materials and Methods:**

An experimental study was conducted, with the response variables being compliance with ergonomic posture requirements (measured using the Compliance Assessment of Dental Ergonomic Posture—CADEP) and quality of cavity preparation (assessed using the Class One Cavity Preparation Assessment—COCA). The independent variable was the magnification level of the Galilean system loupe, tested at four levels: naked eyes, 2.5×, 3.0×, and 3.5×. Second‐year undergraduate dental students (*N* = 40) participated and were divided into Groups I and II. Each student performed cavity preparation at each magnification level, resulting in four preparations per student. Group I started with the naked eye, whereas Group II began with loupes at different magnifications. After 1 week, the conditions were reversed. Working postures were captured in photographs and analysed using CADEP, whereas the quality of the cavity preparations was assessed using COCA. After verifying normality and sphericity, a one‐way repeated measures ANOVA with Bonferroni post hoc test (*α* = 5%) was conducted.

**Results:**

The second results showed that at 3.0× magnification, both compliance with ergonomic posture and the quality of class I cavity preparations were significantly superior compared to the naked eye (*p* < 0.001).

**Conclusions:**

Galilean loupes of 3.0× provided the best balance, improved working posture, and enhanced procedural quality.

## 1 Introduction

A clear view of the operating field, associated with the maintenance of a neutral, ergonomically correct posture, is crucial for enhancing both the productivity and quality of dental work [1]. Various strategies have been employed to achieve this, including the use of ergonomic stools, mouth mirrors for indirect vision, coaxial lighting, and magnification loupes, all of which are becoming increasingly important in dentistry. This growing emphasis on magnification may stem from its ability to improve visualisation of the operating field, facilitating a neutral posture that reduces strain on the arms, back, shoulders, neck, and hands [1, 2].

Early adoption of magnification loupes plays a crucial role in preventing musculoskeletal disorders. During these procedures, both dental students and professionals often focus on work and neglect their posture [3], making loupes particularly beneficial during the training phase. In restorative pre‐clinical training, students continue to refine their skills and typically work within a small operative field that requires precise movement. In this context, magnification devices provide significant advantages by enhancing visual acuity, improving hand‐eye coordination, fostering spatial awareness, and offering a clearer view of the dental field. Hence, these devices not only improve ergonomic posture but also contribute to better visualisation of the teeth [4]. This training phase is critical for encouraging and guiding students in adopting ergonomic work practices [4, 5, 6, 7].

Given that high‐quality loupes involve substantial financial investments [8, 9], careful planning is essential to ensure safe implementation and effective training in educational settings. In particular, the Galilean system loupes are often recommended for those new to magnification, whether students or professionals [1, 3]. This system is not only ergonomically suitable, but also allows for focal distance adjustments and is smaller, lighter, and more affordable than other options [1].

Previous studies have demonstrated that magnification loupes can positively influence dental ergonomics and visual performance. Carpentier et al. [5] found that 2.5× Galilean loupes improved students' posture scores but did not affect the quality of Class II cavity preparations. Similarly, Pazos et al. [1] reported that both Galilean and Keplerian loupes enhanced working posture without significantly impacting the quality of cavity preparations. Wajgarten and Garcia [10] also observed that the use of these systems improved visual acuity and reduced cervical spine deviation among students. More recently, Eggman et al. [11] concluded that 2.5× Galilean loupes enhanced accuracy in performing simpler cavity preparations.

Collectively, the literature support a general consensus that magnification loupes enhance visibility of the operative field, promote ergonomic posture, and may improve fine motor skills and diagnostic ability [5, 8, 9, 10, 11, 12, 13]. However, most investigations have focused on a single magnification level—most commonly 2.5× Galilean systems—and have not explored comparative effects of different magnifications on both posture and procedure quality among novice users. Therefore, scientific evidence remains limited regarding the optimal magnification for improving ergonomic performance and procedural outcomes in inexperienced dental students. This gap underscores the need for the present study, which aimed to assess how different levels of magnification of Galilean loupes influence the working posture and quality of cavity preparation in dental students, thereby contributing valuable data to guide educational strategies and clinical practice.

## 2 Methodology

### 2.1 Ethical Standards

The Research Ethics Committee of the School of Dentistry of São Paulo State University (UNESP), Araraquara Campus (CAAE Registry No. 52497821.4.0000.5416) has approved this experimental study in humans. All the participants provided written informed consent.

### 2.2 Study Design

This experimental study included 40 second‐year undergraduate students from the School of Dentistry of Araraquara, all of whom voluntarily agreed to participate and provided written informed consent. The response variables were compliance with ergonomic posture requirements, measured using the Compliance Assessment of Dental Ergonomic Posture Requirements (CADEP) [14], and the quality of Class I cavity preparations performed by students, evaluated using the Class One Cavity Preparation Assessment (COCA) [15]. The independent variable was the magnification of the Galilean system loupes, which were tested at four levels: naked eye, 2.5×, 3.0×, and 3.5× magnification. Each student completed one cavity preparation at each magnification level, resulting in four preparations per student. Class I cavity preparations for the composite resin were performed on the upper first molar on the operator's working side on a dental mannequin.

The students were randomly assigned to Group I (GI) and Group II (GII), each consisting of 20 participants. Group I began by performing cavity preparations using only the naked eyes, whereas Group II started with the Galilean system loupes with different levels of magnification. After 1 week, the conditions were reversed. To minimise potential bias related to the order of magnification, the sequence was randomised for each student.

### 2.3 Magnification System

In addition to the control condition (naked eyes), the study used binocular loupes from the Galilean headband system at three different magnifications: 2.5×, 3.0×, and 3.5× (Ymarda Optical Instrument Factory, Nanjing, China). The headband system was selected owing to its ability to be fully adjusted to suit different users.

### 2.4 Cavity Preparation

Class I cavities for composite resins were prepared using a 1012 diamond spherical burr at low speed. The burrs were replaced every 10 preparations. A mannequin with artificial resin teeth was used for the procedure. As the teeth were prepared, they were removed and replaced with intact resin teeth to allow for new preparations. The mannequins were attached to a dental chair to simulate a clinical treatment environment. No additional training was provided during the study, as all participants had previously learned and trained in the Class I cavity preparation through the Restorative Dentistry course, ensuring a standardised baseline level of competence for the procedure evaluated.

### 2.5 Recording of Working Postures

Working postures during cavity preparations were recorded using three photographs captured 1 min after the beginning of the procedure by three cameras mounted on tripods at a distance of 1 m from the operator to provide both the lateral and frontal views of each operator.

### 2.6 Postural Assessment

Compliance with ergonomic posture requirements was assessed by a blinded calibrated researcher (*ρ* = 0.969) through visual examination of the images, using the modified CADEP. Each CADEP item was rated as adequate (1 point), partially adequate (0.5 points), or inadequate (0 points). The scores for all items were then summed, with a maximum possible total score of 10.

### 2.7 Assessment of Cavity Preparation Quality

The quality of cavity preparations was assessed by a blinded calibrated researcher (*ρ* = 0.871) using the COCA method [15], which evaluates the following aspects: (1) design, (2) mesiodistal extension, (3) vestibular‐lingual extension, (4) depth, and (5) rounding of internal angles. Each item was scored based on its classification: two points for adequate, one point for partially adequate, and zero for inadequate. At the end of the evaluation, the scores for all items were summed with a maximum possible total score of 10 points. The criteria used in the COCA were aligned with those commonly employed in the academic evaluation of the participating students.

### 2.8 Statistical Analysis

Descriptive statistical analysis was conducted, and after verifying the assumptions of normality and sphericity, a one‐way repeated‐measures Analysis of Variance (ANOVA) with a Bonferroni post‐test was performed. A significance level of 5% was considered significant.

## 3 Results

The mean, standard deviation, and summary of the one‐way repeated‐measures ANOVA of compliance with the ergonomic posture requirements according to the magnification system used are presented in Table 1.

**TABLE 1:** Mean ± SD scores of compliance with ergonomic posture requirements by group.

Magnification system	Mean	Standard deviation
Naked eye	7.57	±1.22A
2.5×	7.97	±1.13AB
3.0×	8.39	±0.87B
3.5×	8.30	±0.79AB

*Note:* One‐way repeated measures ANOVA: *F* = 8.131, *p* < 0.001, *π* = 0.960. Bonferroni post‐test: The same letters indicate statistical similarity.

Compliance with ergonomic posture requirements was significantly higher when using the Galilean 3.0× loupe to perform cavity preparations than when working with the naked eye.

The mean, standard deviation, and summary of the one‐way repeated‐measures ANOVA of the quality of cavity preparations according to the magnification system used are presented in Table 2.

**TABLE 2:** Mean ± SD scores of cavity preparation quality by group.

Magnification system	Mean	Standard deviation
Naked eye	5.02	±0.32A
2.5×	5.52	±0.27AB
3.0×	5.92	±0.28B
3.5×	5.17	±0.22AB

*Note:* One‐way repeated measures ANOVA: *F* = 3.287, *p* = 0.023, *π* = 0.739. Bonferroni post hoc test. The same letters indicate statistical similarity.

It was possible to observe that when using the Galilean 3.0× loupe, the quality of the cavity preparations was significantly superior when compared to work with the naked eye.

## 4 Discussion

This study aimed to evaluate the effect of different magnification levels of Galilean loupes on the working posture of dental students and the quality of their preclinical Class I cavity preparations. The results indicated that the use of the 3.0× Galilean loupe significantly improved compliance with ergonomic posture requirements and the quality of preclinical cavity preparations compared to working without magnification.

To assess working posture, the modified CADEP method was employed, which classifies posture compliance as very low (0–2.5 points), low (2.5–5.0 points), moderate (5.0–7.5 points), and high (7.5–10.0 points). No statistically significant difference was observed between the naked eyes and 2.5 × or 3.5 × magnifications. However, the working posture was classified as moderate when using the naked eyes and as high when using magnification loupes. These findings align with previous research, which also reported the positive effects of magnification loupes on working posture [1, 5, 16, 17, 18].

Regarding the quality of cavity preparations, the superior results observed with the 3.0× magnification loupe may be attributed to its ease of adaptation, offering a larger working field without compromising the focus. In contrast, higher magnifications can lead to a loss of focus owing to small head movements, which can negatively impact posture.

To our knowledge, there are no studies evaluating the effect of different levels of magnification of the Galilean loupe on the quality of procedures. Therefore, the comparison of our results with the literature is limited. Despite this, the studies show positive results regarding 2.5× Galilean loupes. Narula et al. [19] found that cavity preparations performed with those loupes were of higher quality than those performed with the naked eye. They attributed this improvement to the enhanced visibility provided by magnification, which allowed for greater detail in the oral cavity. Similarly, Eggmann et al. [11] reported that undergraduate students demonstrated higher precision when performing simulated cavity preparations using loupes. Additionally, Maggio et al. [20] observed that preclinical students who used magnification loupes developed better psychomotor skills, leading to faster, higher‐quality cavity preparations with less assistance.

The positive effect of the 3.0× magnification loupe on both variables may be linked to the fact that the participants in this study had no prior experience with loupes and did not receive specific training. Therefore, intermediate magnification likely provides a balance between enhanced vision and ease of adaptation. Bud et al. [13] suggested that a moderate level of magnification is ideal during this clinical phase because it offers good visualisation of the operative field without excessively compromising the depth of the field. However, the lack of prior training is a limitation of this study because developing motor skills with magnified vision requires practice. Previous research indicated that operators who regularly use loupes tend to achieve greater precision than those who do not use magnification [11]. This highlights the fact that the full benefits of magnified loupes are best realised after specific training [4, 9, 21, 22].

One limitation of this study is that only a single commercial brand of dental loupes (Ymarda) was tested. This choice was due to the fact that, in Brazil, the use of magnification during undergraduate dental training is not a common educational practice. As a result, participants did not own personal loupes, and the study required a model that could be adjusted to fit multiple users. Ymarda loupes were selected for their fully adjustable design, allowing for standardised use across all students. Additionally, only onecavity preparation per magnification level was evaluated, it is possible that extended use of the loupes over an entire clinical session or multiple procedures might have produced different results.

Based on the findings of this and other studies [1, 5, 11, 16, 23, 24], the adoption of magnification in clinical practice offers more advantages than disadvantages. It is likely to become the standard of care in dentistry shortly [13, 21]. Clinical dentistry, regardless of the specialty, presents numerous challenges, but having optimal vision and working in a comfortable posture can significantly enhance the clinical experience of both professionals and patients [13]. Therefore, the introduction of magnified loupes during the training stage is highly beneficial for the dental community. Dental schools worldwide should incorporate the use of magnified vision into their curricula to foster more ergonomic, efficient, and higher‐quality dental practices [4, 25].

## 5 Conclusion

The use of the 3.0× Galilean magnification loupe was found to significantly improve compliance with ergonomic posture requirements and enhance the quality of Class I cavity preparations compared with working without magnification.

## Conflicts of Interest

The authors declare no conflicts of interest.

## Data Availability

The data that support the findings of this study are available from the corresponding author upon reasonable request.
